# “Barriers" to Child Development and Human Potential: The Case for Including the “Neglected Enteric Protozoa" (NEP) and Other Enteropathy-Associated Pathogens in the NTDs

**DOI:** 10.1371/journal.pntd.0002125

**Published:** 2013-04-11

**Authors:** Luther A. Bartelt, Aldo A. M. Lima, Margaret Kosek, Pablo Peñataro Yori, Gwenyth Lee, Richard L. Guerrant

**Affiliations:** 1 Center for Global Health and Division of Infectious Disease, University of Virginia, Charlottesville, Virginia, United States of America; 2 Department of Physiology and Pharmacology, Institute of the Brazilian Semi-Arid, School of Medicine, Federal University of Ceara, Fortaleza, Brazil; 3 Johns Hopkins School of Public Health and Hygiene, Baltimore, Maryland, United States of America; National Travel Health Network and Centre, United Kingdom (retired)

The World Health Organization (WHO) has set forth ambitious efforts to control, and where possible, eliminate the neglected tropical diseases (NTDs) that contribute to poverty and “impair the ability of those infected to achieve their full potential, both developmentally and socio-economically" [1], [2]. This neglected disease initiative's (NDI) purpose has been to close the existing poverty gap between individuals living in low/middle-income and high-income countries, and thus facilitate the achievement of the 2000 Millennium Developmental Goals [3]. The gap is still large. Yet, some marked achievements of the NDI, including coordinated administration of preventive chemotherapy to nearly 670 million children globally and the imminent elimination of dracunculiasis, give hope that the WHO's NTD paradigm, a “five-pronged" approach of 1) preventive chemotherapy, 2) intensified case-management, 3) vector control, 4) provision of safe water, sanitation, and hygiene, and 5) veterinary public health, are proving beneficial [1].

Malnutrition and unfulfilled human potential are widely prevalent among the 1.4 billion people also afflicted by the principal NTDs. Over the last decade, we have become increasingly aware that alterations in intestinal function not only associate with malnutrition, but are likely one of its driving forces. It was recognized half a century ago that children in developing countries had intestinal mucosa that showed morphological flattening [4] and malabsorption [5] that were reversible upon exposure to a cleaner environment. Similarly, Lindenbaum also showed in the 1960s that Peace Corps volunteers with diarrhea and malnutrition had biochemical markers of malabsorption: 40% had decreased d-xylose levels, and 52% had low Schilling tests. Moreover, 88% of intestinal biopsies from these volunteers showed mild to moderate jejunitis with decreased villus∶crypt ratios [6].

Villus blunting along with chronic inflammation associates with impaired intestinal barrier function with resultant increased intestinal permeability [7]. This combination of altered villus architecture and barrier function, so uniquely dependent upon one's environment, has been termed “environmental enteropathy" (EE) [8]. These characteristic EE changes have been epidemiologically linked with growth faltering and are one hypothesis for why intensive nutritional supplementation interventions done under even ideal clinical trial conditions have significant but limited success in improving weight, linear growth, and cognitive function [9]–[11].

Syndemic with environmental enteropathy are high rates of childhood diarrhea. Although children may display “catch-up" growth following isolated and short-lived (3 days) diarrheal episodes [12], prolonged and persistent diarrheal (>14 days) episodes strongly associate with stunting [13]–[16]. Testament to the effectiveness of WHO campaigns, the combination of oral rehydration solution (ORS) in the 1980s and its subsequent refinements, and the introduction of rotavirus vaccines in some populations [17], have led to significant reductions in diarrhea-related mortality from 4.5 million/year over a decade ago to 1.5 million/year in 2010 [18], [19]. Currently, however, there are still >700,000 deaths per year globally related to diarrheal diseases [20], and conversely, the frequency of diarrheal episodes has not declined but remains unacceptably high [21]. Among the myriad pathogens causing diarrhea in low/middle-income countries, the protozoa *Giardia lamblia* (synonymous with *G. intestinalis/G. duodenalis*) and *Cryptosporidium* spp. are among the most commonly isolated [22]. Although present in *PLOS NTDs'* expanded NTD list [23], and added to the WHO's NDI in 2004 [2], [24], these organisms are not mentioned in the 2010 WHO NTD report [1]. Despite the call for increased surveillance [2], the true global prevalence of these infections remains poorly defined [25]. In the past few years, we have learned important lessons that make imperative an emphasis on these “neglected enteric protozoa" (NEP) and other enteropathy-associated pathogens within the NDI:

1. The enteric protozoa should be classified with the soil-transmitted helminths (STHs) as pathogens associated with stunting.
2. We need to restructure our theoretical framework to broaden our concept of “diarrheal" disease to include “asymptomatic" enteric infections.
3. We must recognize that environmental enteropathy is likely both a common and complex disease, and that infections related to the development or exacerbation of environmental enteropathy deserve prioritization in disease control strategies.
4. Therapeutic strategies against enteropathy-associated pathogens can and should be evaluated for efficacy within the existing NDI programs.

## The NEP Belong with the STHs as Stunting Pathogens

The NEP *Cryptosporidium*, *E. histolytica*, and *G. lamblia* (as well as other enteropathogens such as enteroaggregative *E. coli* [EAEC]) are increasingly recognized to be associated with growth shortfalls [26]–[36]. Mondal et al. recently demonstrated that stunted infants in Bangladesh were at increased risk of severe diarrhea, and in particular, diarrhea-associated *E. histolytica* and *Cryptosporidium* infections [24]. The impact of enteric protozoal infections has a wide geographic distribution with specific associations between *Cryptosporidium* and malnutrition in Jamaica [27], Israel [28], Peru [29], Mexico [30], Uganda [31], Bangladesh [22], [32], [33], and Brazil [34]–[36]. Furthermore, both *Cryptosporidium* and variably *Giardia* associate with persistent diarrhea [14], [37]. Additionally, we have found that even when controlled for nutritional status, early childhood cryptosporidial infection either with or without diarrhea was associated with reduced fitness at 6–9 years of age and retarded weight gain [37]. Also, similar to the cognitive deficits seen in infestation with STHs [38], *Giardia* has been associated with decreased cognitive performance [39]–[41]. The overall impact of even “asymptomatic" disease from enteric protozoa, therefore, may be a major contributor to lost human potential. Moreover, the incoming field data from large multicenter trials such as the Global Enterics Multicenter Study (GEMS) [42] are demonstrating a morass of diverse enteric infections. Such infections occur both concomitantly and sequentially with STHs. It is quite plausible that along with their individual impacts, there is a potentially synergistic effect between STHs and NEP whereby sequential hits affect children at their most vulnerable ages and serially interrupt crucial developmental milestones. The growth impairments that directly result from *Cryptosporidium* and other enteropathogens (such as EAEC) are clear not only from these field studies, but also from animal models in which the “vicious cycle" of enteric infection and malnutrition can be causally linked [43]–[45]. Future investigations assessing the long-term, and possibly permanent, impact of these serial exposures into adulthood could be included in the assessments of STH outcomes through NDI surveillance.

## We Need to Restructure Our Conceptualization beyond Diarrheal Disease to Enteropathy

The NEP may be signaling us to expand our understanding of “diarrheal" disease. Beginning with the pioneering work of L. Mata, who showed that repeated episodes of diarrheal disease were temporally linked with associated linear growth shortfalls [46], to the observations associating even “asymptomatic" enteric protozoa infections with developmental impairments, we have been challenged to re-conceptualize our traditional case definition of “diarrheal" disease. The GEMS findings presented at the recent American Society of Tropical Medicine and Hygiene annual meeting revealed that *Cryptosporidium* ranks highly (in some populations, second only to rotavirus) among all viral, bacterial, and parasitic pathogens causing moderate to severe diarrhea in children, and that the parasite also associates with persistent linear growth shortfalls [42]. The GEMS investigators also recently published a systematic review and meta-analysis demonstrating that *Giardia*, present in >90% of children in some populations by 12 months of age [22], was strongly associated with persistent diarrhea, but inversely associated with acute diarrhea in endemic settings [47]. Elsewhere, *Giardia* associates with wasting (WAZ<−2) [48], [49] and stunting [50], [51], suggesting the parasite (or particular *G. lamblia* strains) may have an underappreciated influence on childhood development. Thus, as we had discovered with the more readily identifiable STHs using stool microscopy, we need to examine not only the overt liquid diarrhea, but also developmental shortfalls that may indicate a more silent and ominous threat to human potential. Indeed, restructured key “case" definitions of disease resulting from enteric infections will be critical to correctly determining the true prevalence and impact of these infections, and thus to prioritizing treatment and prevention strategies in the most appropriate manner.

## Environmental Enteropathy Is Likely both Common and Complex

The mechanisms accounting for growth impairments following infection are complex, and they are likely intertwined with environmental enteropathy. There is a pathological basis for the NEP to potentially initiate and/or propagate environmental enteropathy. In murine models, *Cryptosporidium* causes weight loss, villus blunting, crypt hyperplasia, and increased IFN-gamma and TNF-alpha reminiscent of EE changes [44], [52]. Chronic *G. lamblia* infection in humans has also demonstrated abnormalities in epithelial tight-junction proteins (claudin-1) and altered mucosal morphometry [53]. A better understanding of the pathogenesis of the NEP and EE and their developmental sequlae may help to identify common pathways by which certain enteric pathogens, together with malnutrition, promote lost human potential. Such discoveries may open avenues for new therapeutics that restore gut function. Despite the aforementioned foundational observations linking malnutrition, enteric infections, and alterations in intestinal architecture, inflammation, and function, it has taken many years to begin to dissect the etiologies and mechanisms driving EE. One significant hurdle to overcome is the challenge inherent in studying a disease process that otherwise requires endoscopy and tissue biopsy for diagnosis. The pursuit for reliable non-invasive biomarkers of EE that are readily available and both sensitive and specific is crucial for determining the true prevalence of this condition, and for increasing our understanding of its underlying pathophysiology and disease modulators (i.e., differential effects from various pathogens and co-pathogen infections, nutritional status and micronutrient intake, host genetics/epigenetics, and microbiota). Studies are currently investigating several leading candidate biomarkers found to be elevated in children in low-income countries, including: markers of intestinal inflammation such as fecal myeloperoxidase (MPO), lactoferrin, and neopterin; serum alpha-1 anti-trypsin (A1AT), a marker of hyperpermeability and protein wasting; and serum endotoxin core antibody (EndoCAb), a marker of bacterial translocation and systemic immune activation. Clinical Investigations in this field have identified that elevated fecal lactoferrin and EndoCAb are present in malnourished children and in certain enteric infections, and that malnourished children have increased lactulose∶mannitol ratios (L∶M) [22], [54]. A combination of these candidate biomarkers or novel approaches such as metabonomics are needed to begin to truly appreciate the global prevalence, spectrum, and impact of EE and the differential influence NEP and other pathogens have on its severity.

## Strategies toward Reducing the Burden of the NEP Can Be Incorporated into Existing NDI Programs

**Figure 1:**
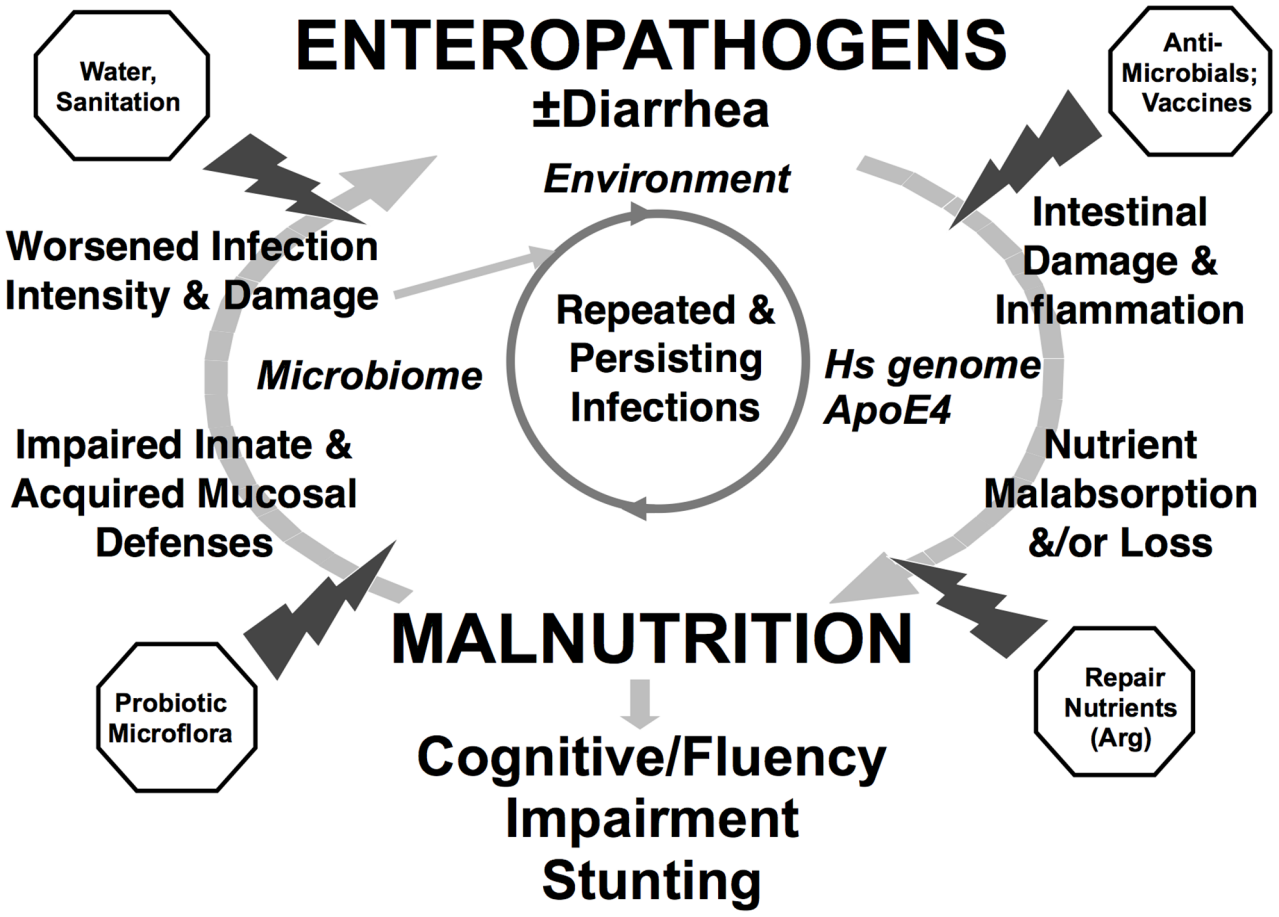
The “vicious cycle" of enteropathogens, malnutrition, and impaired childhood development, and multifaceted opportunities for intervention. Figure adapted from Nutr Rev. 2008 September; 66(9): 487–505 [15].

Even when not associated with diarrhea, the capacity of the NEP and other enteropathy-associated pathogens to limit human potential in populations living in poverty, and the increasing recognition of the systemic effects of EE, suggest that novel strategies are needed to fully address the burden of enteric pathogens. Though provision of safe water and sanitation should be universal, not all water purification techniques are effective against the chlorine-resistant and environmentally hardy protozoa such as *Cryptosporidium* spp. Therapeutic strategies are also needed. As was recently demonstrated, mass azithromycin distribution for trachoma was associated with reduced all-cause mortality and infectious childhood mortality [55], a benefit that could be partially attributed to decreased intestinal pathogen burden [56]. Albendazole given in a 5-day regimen has similar efficacy to metronidazole for *Giardia* [57], which could be incorporated into mass de-worming campaigns with extended therapy for *Giardia*-endemic regions. The addition of nitazoxanide as an anti-protozoal agent, and more specifically for *Cryptosporidium* [58], may synergize with azithromycin and albendazole in mass preventive chemotherapy campaigns. Novel intestinal repair therapies that could be added to the presently recommended zinc supplementation need to be identified and incorporated as measures to combat the “vicious malnutrition-enteric disease cycle" (Figure 1 [15]) [59], [60].

We have here enumerated several reasons why increased awareness of NEP and other pathogens associated with changes resembling EE is necessary to achieve the NDI's goals of eliminating diseases of poverty. Environmental enteropathy, so intimately dependent upon individuals' surroundings, is perhaps our closest physiopathological correlate to poverty. Through its multi-pronged approach, including intensified case recognition and management, the NDI has the existing infrastructure to take over where decades of acute life-saving oral rehydrating therapy has left off, and begin to reverse the trends of increasing diarrhea-associated morbidities [21]. Alongside efforts to combat STHs, we need aggressive measures to address “stunting" and “wasting" enterics such as *Cryptosporidium*, *E. histolytica, G. lamblia*, and other pathogens (i.e., EAEC) as they are identified. Such measures will prove critical for the more than one-third of the world's children among the “bottom billion" to achieve their full human potential.

## References

[1] Savioli LS, Daumerie D (2010) First WHO report on neglected tropical diseases: working to overcome the global impact of neglected tropical diseases. Geneva: World Health Organization. pp. 1–169.

[2] Savioli L, Smith H, Thompson A (2006) *Giardia* and *Cryptosporidium* join the ‘Neglected Diseases Initiative’. Trends Parasitol 22: 203–208. 16545611 10.1016/j.pt.2006.02.015

[3] Hotez PJ, Molyneux DH, Fenwick A, Kumaresan J, Sachs SE, et al (2007) Control of neglected tropical diseases. N Engl J Med 357: 1018–1027. 17804846 10.1056/NEJMra064142

[4] Schenk EA, Samlof IM, Klipstein FA (1968) Morphology of small bowel biopsies. Am J Clin Nutr 21: 944–961. 5675858 10.1093/ajcn/21.9.944

[5] Lindenbaum J, Harmon JW, Gerson CD (1972) Subclinical malabsorption in developing countries. Am J Clin Nutr 25: 1056–1061. 4562265 10.1093/ajcn/25.10.1056

[6] Lindenbaum J, Kent TH, Sprinz H (1966) Malabsorption and jejunitis in American Peace Corps volunteers in Pakistan. Ann Intern Med 65: 1201–1209. 5928480 10.7326/0003-4819-65-6-1201

[7] Menzies IS, Zuckerman MJ, Nukajam WS, Somasundaram SG, Murphy B, et al (1999) Geography of intestinal permeability and absorption. Gut 44: 483–489. 10075954 10.1136/gut.44.4.483 PMC1727437

[8] Salazar-Lindo E, Allen S, Brwester DR, Elliott EJ, Fasano A, et al (2004) Intestinal infections and environmental enteropathy: Working Group report of the Second World Congress of Pediatric Gastroenterology, Hepatology and Nutrition. J Pediatr Gastroenterol Nutr 39: S662–S669. 15184767 10.1097/00005176-200406002-00013

[9] Campbell DI, Elia M, Lunn PG (2003) Growth faltering in rural Gambian infants is associated with impaired small intestinal barrier function, leading to endotoxemia and systemic inflammation. J Nutr 133: 1332–1338. 12730419 10.1093/jn/133.5.1332

[10] Lunn PG, Northrop-Clewes CA, Downes RM (1991) Intestinal permeability, mucosal injury, and growth faltering in Gambian infants. Lancet 338: 907–910. 1681266 10.1016/0140-6736(91)91772-m

[11] Dewey KG, Adu-Afarwuah S (2008) Systematic review of the efficacy and effectiveness of complementary feeding interventions in developing countries. Matern Child Nutr 4 Suppl 1: 24–85. 18289157 10.1111/j.1740-8709.2007.00124.x PMC6860813

[12] Briend A, Hasan KZ, Aziz KM, Hoque BA (1989) Are diarrhea control programmes likely to reduce malnutrition? Observations from rural Bangladesh. Lancet 2: 319–322. 2569114 10.1016/s0140-6736(89)90498-4

[13] Lima AA, Guerrant RL (1992) Persistent diarrhea in children: epidemiology, risk factors, pathophysiology, nutritional impact, and management. Epidemiol Rev 14: 222–242. 1289113 10.1093/oxfordjournals.epirev.a036088

[14] Lima AA, Moore SR, Barboza MS, et al (2000) Persistent diarrhea signals a critical period of increased diarrhea burdens and nutritional shortfalls: a prospective cohort study among children in northeastern Brazil. J Infect Dis 181: 1643–1651. 10823764 10.1086/315423

[15] Guerrant RL, Oriá RB, Moore SR, Oriá MO, Lima AA (2008) Malnutrition as an enteric infectious disease with long-term effects on child development. Nutr Rev 66: 487–505. 18752473 10.1111/j.1753-4887.2008.00082.x PMC2562291

[16] Moore SR, Lima NL, Soares AM, et al (2010) Prolonged episodes of acute diarrhea reduce growth and increase risk of persistent diarrhea in children. Gastroenterology 139: 1156–1164. 20638937 10.1053/j.gastro.2010.05.076 PMC2949449

[17] do Carmo GMI, Yen C, Cortes J, Siqueira AA, de Oliveira WK, et al (2011) Decline in Diarrhea Mortality and Admissions after Routine Childhood Rotavirus Immunization in Brazil: A Time-Series Analysis. PLoS Med 8 (4) e1001024 doi:10.1371/journal.pmed.1001024. 21526228 10.1371/journal.pmed.1001024 PMC3079643

[18] Black RE, Cousens S, Johnson HL, et al (2010) Global, regional, and national causes of child mortality in 2008: a systematic analysis. Lancet 375: 1969–1987. 20466419 10.1016/S0140-6736(10)60549-1

[19] World Health Organization (2012) Diarrhoeal disease. Available: http://www.who.int/mediacentre/factsheets/fs330/en/index.html. Accessed 2012 January 15.

[20] Liu L, Johnson HL, Cousens S, Perin J, Scott S, Lawn JE, Rudan I, Campbell H, Cibulskis R, Li M, Mathers C, Black RE Childh Health Epidemiology Reference Group of WHO and UNICEF (2012) Global, regional, and national causes of child mortality: an updated systematic analysis for 2010 with time trends since 2000. Lancet 379 (9832) 2151–2161. 22579125 10.1016/S0140-6736(12)60560-1

[21] Kosek M, Bern C, Guerrant RL (2003) The global burden of diarrhoeal disease, as estimated from studies published between 1992 and 2000. Bull World Health Organ 81: 197–204. 12764516 PMC2572419

[22] Mondal D, Minak J, Alam M, Liu Y, Dai J, et al (2012) Contribution of enteric infection, altered intestinal barrier function, and maternal malnutrition to infant malnutrition in Bangladesh. Clin Infect Dis 54: 185–192. 22109945 10.1093/cid/cir807 PMC3245731

[23] Hotez PJ, Pecoul B (2010) Manifesto for advancing the control and elimination of neglected tropical diseases. PLoS Negl Trop Dis 4: e718 doi:10.1371/journal.pntd.0000718. 20520793 10.1371/journal.pntd.0000718 PMC2876053

[24] World Health Organization/CS/IPI/92.2 (1991) WHO/PAHO Informal consultation on intestinal protozoal infections, Mexico.

[25] Bethony JM, Cole RN, Guo X, Kamhawi S, Lightowlers MW, et al (2011) Vaccines to combat the neglected tropical diseases. Immunol Rev 239: 237–270. 21198676 10.1111/j.1600-065X.2010.00976.x PMC3438653

[26] Guerrant RL, Oriá RB, Moore SR, Oriá MO, Lima AA (2008) Malnutrition as an enteric infectious disease with long-term effects on child development. Nutr Rev 66: 487–505. 18752473 10.1111/j.1753-4887.2008.00082.x PMC2562291

[27] Macfarlane DE, Horner-Bryce J (1987) Cryptosporidiosis in well-nourished and malnourished children. Acta Paediatr 76: 474–477. 10.1111/j.1651-2227.1987.tb10502.x 3604664

[28] Sallon S, Deckelbaum RJ, Schmid II, Harlap S, Baras M, et al (1988) *Cryptosporidium*, malnutrition, and chronic diarrhea in children. Am J Dis Child 142: 312–315. 3344720 10.1001/archpedi.1988.02150030086027

[29] Sarabia-Arce S, Salazar-Lindo E, Gilman RH, Naranjo J, Miranda E (1990) Case-control study of *Cryptosporidium parvum* infection in Peruvian children hospitalized for diarrhea: possible association with malnutrition and nosocomial infection. Pediatr Infect Dis J 9: 627–631. 2235186

[30] García VE, Chávez LM, Coello RP, González J, Aguilar BS (1991) *Cryptosporidium sp* in 300 children with and without diarrhea. Arch Invest Med (Mex) 22: 329–332. 1844120

[31] Tumwine JK, Kekitiinwa A, Nabukeera N, Akiyoshi DE, Rich SM, et al (2003) *Cryptosporidium parvum* in children with diarrhea in Mulago Hospital, Kampala, Uganda. Am J Trop Med Hyg 68: 710–715. 12887032

[32] Mondal D, Haque R, Sack RB, Kirkpatrick BD, Petri WA (2009) Attribution of malnutrition to cause-specific diarrheal illness: evidence from a prospective study of preschool children in Mirpur, Dhaka, Bangladesh. Am J Trop Med Hyg 80: 824–826. 19407131 PMC3410540

[33] Haque R, Mondal D, Karim A, Hossain Molla I, Rahim A, et al (2009) Prospective case-control study of the association between common enteric protozoal parasites and diarrhea in Bangladesh. Clin Infect Dis 48: 1191–1197. 19323634 10.1086/597580 PMC2883291

[34] Guerrant DI, Moore SR, Lima AA, Patrick PD, Schorling JB, et al (1999) Association of early childhood diarrhea and cryptosporidiosis with impaired physical fitness and cognitive function four-seven years later in a poor urban community in northeast Brazil. Am J Trop Med Hyg 61: 707. 10586898 10.4269/ajtmh.1999.61.707

[35] Newman RD, Moore SR, Lima AA, Nataro JP, Guerrant RL, et al (2001) A longitudinal study of *Giardia lamblia* infection in Northeast Brazilian children. Trop Med Int Health 6: 624–634. 11555428 10.1046/j.1365-3156.2001.00757.x

[36] Agnew DG, Lima AA, Newman RD, Wuhib T, Moore RD, et al (1998) Cryptosporidiosis in northeastern Brazilian children: association with increased diarrhea morbidity. J Infect Dis 177: 754–760. 9498458 10.1086/514247

[37] Checkley W, Epstein LD, Gilman RH, Black RE, Cabrera L, et al (1998) Effects of *Cryptosporidium parvum* infection in Peruvian children: growth faltering and subsequent catch-up growth. Am J Epidemiol 148: 497–506. 9737562 10.1093/oxfordjournals.aje.a009675

[38] Gilman RH, Chong YH, Davis C, Greenberg B, Virik HK, et al (1983) The adverse consequences of heavy *Trichuris* infection. Trans R Soc Trop Med Hyg 77: 432–438. 6636270 10.1016/0035-9203(83)90103-7

[39] Berkman DS, Lescano AG, Gilman RH, Lopez SL, Black MM (2002) Effects of stunting, diarrhoeal disease, and parasitic infection during infancy on cognition in late childhood: a follow-up study. Lancet 359: 564–571. 11867110 10.1016/S0140-6736(02)07744-9

[40] Ajjampur SS, Koshy B, Venkataramani M, Sarkar R, Joseph AA, et al (2011) Effect of cryptosporidial and giardial diarrhoea on social maturity, intelligence and physical growth in children in a semi-urban slum in south India. Ann Trop Paediatr 31: 205–210. 21781414 10.1179/1465328111Y.0000000003

[41] Guerrant RL, Oria RB, Moore SR, Scharf R, Lima AAM (2011) Enteric protzoa and human potential. Ann Trop Paediatr 31: 2 01–203. 10.1179/146532811X13006353133911 PMC3296476 21781413

[42] Kotloff LK (2011) “Top 5" attributable pathogens of moderate and severe diarrhea (by age, study site and clinical presentation) and mortality and linear growth consequences. 60^th^ Annual Meeting of the American Socity of Tropical Medicine and Hygiene. Philadelphia, PA.

[43] Coutinho BP, Oriá RB, Vieira CM, Sevilleja JE, Warren CA, et al (2008) Cryptosporidium infection causes undernutrition and, conversely, weanling undernutrition intensifies infection. J Parasitol 94: 1225–1232. 18576767 10.1645/GE-1411.1 PMC3070954

[44] Costa LB, JohnBull EA, Reeves JT, Sevilleja JE, Freire RS, et al (2011) Cryptosporidium-malnutrition interactions: mucosal disruption, cytokines, and TLR signaling in a weaned murine model. J Parasitol 6: 1113–1120. 10.1645/GE-2848.1 PMC3247658 21711105

[45] Roche JK, Cabel A, Sevilleja J, Nataro J, Guerrant RL (2010) Enteroaggregative escherichia coli (EAEC) impairs growth while malnutrition worsens EAEC infection: A novel murine model of the infection malnutrition cycle. J Infect Dis 202 (4) 506–514. 20594107 10.1086/654894 PMC2919845

[46] Mata LJ (1978) The children of Santa Maria Cauque: A prospective field study of health and growth. Cambridge, MA: MIT Press.

[47] Muhsen K, Levine M (2012) A systematic review and meta-analysis of the asssociation between *G. lamblia* and endemic pediatric diarrhea in developing countries. Clin Inf Dis 55 (4) S271–S293. 10.1093/cid/cis762 PMC3502312 23169940

[48] Al-Mekhlafi MS, Azlin M, Nor Aini U, Shaik A, Sa'iah A, et al (2005) Giardiasis as a predictor of childhood malnutrition in Orang Asli children in Malaysia. Trans R Soc Trop Med Hyg 99: 686–691. 15992838 10.1016/j.trstmh.2005.02.006

[49] Carvalho-Costa FA, Gonçalves AQ, Lassance SL, Silva Neto LM, Salmazo CA, et al (2007) *Giardia lamblia* and other intestinal parasitic infections and their relationships with nutritional status in children in Brazilian Amazon. Rev Inst Med Trop Sao Paulo 49: 147–153. 17625691 10.1590/s0036-46652007000300003

[50] Gupta MC, Urrutia JJ (1982) Effect of periodic antiascaris and antigiardia treatment on nutritional status of preschool children. Am J Clin Nutr 36: 79–86. 7091037 10.1093/ajcn/36.1.79

[51] Botero-Garcés JH, García-Montoya GM, Grisales-Patiño D, Aguirre-Acevedo DC, Alvarez-Uribe MC (2009) *Giardia intestinalis* and nutritional status in children participating in the complementary nutrition program, Antioquia, Colombia, May to October 2006. Rev Inst Med Trop Sao Paulo 51: 155–162. 19551290 10.1590/s0036-46652009000300006

[52] Costa LB, Noronha FJ, Roche JK, Sevilleja JE, Warren CA, et al (2012) Novel in vitro and in vivo models and potential new therapeutics to break the vicious cycle of Cryptosporidium infection and malnutrition. J Inf Dis 9: 1464–1471. 10.1093/infdis/jis216 PMC3324401 22454464

[53] Troeger H, Eppie HJ, Schneider T, Wahnschaffe U, Ullrich R, et al (2007) Effect of chronic *Giardia lamblia* infection on epithelial transport and barrier function in human duodenum. Gut 56: 328–335. 16935925 10.1136/gut.2006.100198 PMC1856804

[54] Barboza Junior MS, Silva TM, Guerrant RL, Lima AA (1999) Measurement of intestinal permeability using mannitol and lactulose in children with diarrheal diseases. Braz J Med Biol Res 32: 1499–1504. 10585631 10.1590/s0100-879x1999001200008

[55] Keenan JD, Ayele B, Gebre T, Zerihun M, Zhou Z, et al (2011) Childhood mortality in a cohort treated with mass azithromycin for trachoma. Clin Inf Dis 52: 883–888. 10.1093/cid/cir069 PMC3106233 21427395

[56] Guerrant RL, Bartelt LA, Scharf RJ (2012) Thinking deeper about important mass treatment trials. Clin Inf Dis 54: 1674–1675. 10.1093/cid/cis241 PMC3404721 22431805

[57] Solaymani-Mohammadi S, Genkinger JM, Loffredo CA, Singer SM (2010) A meta-analysis of the effectiveness of albendazole compared with metronidazole as treatments for infections with Giardia duodenalis. PLoS Negl Trop Dis 4: e682 doi:10.1371/journal.pntd.0000682. 20485492 10.1371/journal.pntd.0000682 PMC2867942

[58] Rossignol JF, Kabil SM, el-Gohary Y, Younis AM (2006) Effect of nitazoxanide in diarrhea and enteritis caused by Cryptosporidium species. Clin Gastroenterol Hepatol 4: 320–324. 16527695 10.1016/j.cgh.2005.12.020

[59] Lima AA, Brito LF, Ribeiro HB, Martins MC, Lustosa AP, et al (2005) Intestinal barrier function and weight gain in malnourished children taking glutamine supplemented enteral formula. J Pediatr Gastroenterol Nutr 40: 28–35. 15625423 10.1097/00005176-200501000-00006

[60] Lima NL, Soares AM, Mota RM, Monteiro HS, Guerrant RL, et al (2007) Wasting and intestinal barrier function in children taking alanyl-glutamine-supplemented enteral formula. J Pediatr Gastroenterol Nutr 44: 365–374. 17325559 10.1097/MPG.0b013e31802eecdd

